# Up-regulation of peroxiredoxin-1 promotes cell proliferation and metastasis and inhibits apoptosis in cervical cancer

**DOI:** 10.7150/jca.37147

**Published:** 2020-01-01

**Authors:** Ermei Lu, Xiaoli Hu, Chunyu Pan, Jingjing Chen, Yichi Xu, Xueqiong Zhu

**Affiliations:** Department of Obstetrics and Gynecology, the Second Affiliated Hospital of Wenzhou Medical University, Wenzhou 325027, China.

**Keywords:** PRDX1, cervical cancer, proliferation, migration, invasion, apoptosis

## Abstract

**Objective:** To investigate the effect of peroxiredoxin 1 (PRDX1) on the biological behavior of cervical cancer cells and the possible mechanism.

**Materials and methods:** The expression of PRDX1 in human cervical cancer tissues and adjacent non-tumor tissues were detected by immunohistochemistry (IHC). Lentivirus containing PRDX1-cDNA or shRNA against PRDX1 was constructed to overexpress or knockdown PRDX1 in SiHa cervical cancer cells. Cell proliferation was tested by CCK-8 and BrdU incorporation assay and cell apoptosis was evaluated by AnnexinV-PE /7AAD assay. Scratch wound and transwell invasion assay were used to test migration and invasion activity after PRDX1 was overexpressed or suppressed. Furthermore, the effect of PRDX1 on cell proliferation and apoptosis was also studied using a xenograft model of nude mice.

**Results:** The expression of PRDX1 protein was significantly up-regulated in the tumor tissues compared with the paired adjacent non-tumor tissues. Meanwhile, PRDX1 overexpression was associated with tumor stage, lymphatic metastasis and differentiation. Overexpression of PRDX1 significantly promoted proliferation and inhibited apoptosis by increasing the expression of Nanog, proliferating cell nuclear antigen (PCNA), B-cell lymphoma-2 (Bcl-2) and downregulating the expression of Bcl2-associated X protein (BAX) in SiHa cervical cancer cells. Moreover, PRDX1 overexpression increased invasion and migration of SiHa cervical cancer cells via up-regulating the expression of Snail and matrix metalloprotein 9 (MMP-9) and down-regulating the expression of E-cadherin. Knockdown of PRDX1 resulted in the opposite results. The role of PRDX1 in promoting SiHa cervical cancer cell proliferation and inhibiting apoptosis has also been confirmed *in vivo* in a mouse xenograft model.

**Conclusions:** PRDX1 promoted cell proliferation, migration, and invasion and suppressed apoptosis of cervical cancer possibly via regulating the expression of related protein.

## Introduction

Cervical cancer, the most common gynecological cancers all over the world, remains a leading cause of cancer-related death for women in developing countries. Cervical cancer is the second leading cause of cancer death in women aged 20 to 39 years 1. According to the statistics, 570,000 women were diagnosed with cervical cancer, and 311,000 patients died of the disease in 2018 worldwide 2. Seventy-five percent of women living in resource-poor countries who are diagnosed with cervical cancer present with locally advanced disease 3. The 5-year survival rate for women with metastatic cervical cancer is only 16.5% 4. According to clinical staging, cervical cancer is usually treated by surgery, radiation or chemotherapy. Early-stage disease is mainly treated by radical surgery, which usually harvests good clinical efficacy. The recommended treatment for locally advanced cervical cancer is the combination of chemotherapy and radiation 5. However, the side effects caused by radiotherapy and chemotherapy often seriously affect the quality of life of patients. Therefore, there is an urgent need to explore the underlying molecular mechanisms of cervical cancer proliferation and metastasis.

Peroxiredoxins (PRDXs) are a family of peroxidase enzymes, which are expressed in a variety of organizations, and perform multiple important functions by regulating peroxide levels within cells. PRDX family consists of six isoforms in human, namely peroxiredoxin 1, 2, 3, 4, 5, and 6 6. Based on cysteine residues participated in catalysis, PRDXs are divided into two subgroups: 2-Cys, and 1-Cys^7^. PRDXs exert its protective function by breaking down hydrogen peroxide, organic hydroperoxides and peroxynitrite^7^. PRDXs can also protect the protein from oxidative injury or degradation by acting as a molecular chaperone^8^. Recently, studies have shown that PRDXs play an important role in the regulation of various cellular processes, including proliferation, differentiation, and apoptosis^9^. Studies demonstrate that PRDXs are involved in cancers, inflammatory related diseases and neurodegenerative diseases 10. Since PRDXs can eliminate reactive oxygen species generated by the rapidly dividing tumor cells, and the expression changes of this family of proteins are most likely to affect tumor cell proliferation significantly 11.

Recently, studies have shown that PRDX1 is up-regulated in several human cancers types, including lung 12, breast 13, hepatocellular 14, colorectal 15, gastric 16, esophagus 17, prostate 18, ovarian 19 and pancreatic cancer 20. PRDX1 knockout mice acquire severe hemolytic anemia and are more susceptible to the development of several cancers 21. PRDX1 has also been proven to be capable of inhibiting tumorigenesis through suppressing H-Ras and ErbB-2-induced transformation which was mainly achieved by promoting the activity of phosphatase and tensin homolog (PTEN) 22. In our previous study, we detected expression changes of proteins in cervical cancer tissues before and after neoadjuvant chemotherapy (NAC) by using mass spectrometry, and found that PRDX1 was significantly increased after NAC treatment 23. The above results suggest that the expression of PRDX1 may be involved in cervical cancer. However, the expression of PRDX1 in cervical cancer tissues is still unclear. Besides, the impacts of PRDX1 expression on tumor cell proliferation, differentiation, and apoptosis have rarely been investigated in human cervical cancer.

On account of the lack of understanding about the role that PRDX1 played in cervical cancer, further study should be carried out to elucidate its function. In this study, the lentiviral vector containing PRDX1-cDNA or PRDX1-shRNA was used to up-regulate or down-regulate the expression of PRDX1 in cervical cancer cells, and the functional roles of PRDX1 on the biological behavior of cervical cancer were investigated. Additionally, the xenograft model was established to evaluate whether PRDX1 affects the growth of cervical cancer *in vivo,* and proliferation index and apoptosis index in tumor tissues were assessed by the TdT-mediated dUTP nick end labeling (TUNEL) assay and PCNA immunohistochemical staining.

## Materials and Method

### Patients and specimens

All tissue samples from cervical cancer patients were collected by surgical excisions resection between 2014 and 2016 at Second Affiliated Hospital of Wenzhou Medical University. A total of 20 formalin-fixed paraffin-embedded tissues including paired tumor and adjacent non-tumor tissues were collected and identified by three experienced pathologists before IHC staining. None of the patients received chemotherapy or radiotherapy before specimen collection. The study was approved by the ethics committee of the Second Affiliated Hospital of Wenzhou Medical University, and all patients were provided with written informed consent.

### Gene expression profiling interactive analysis (GEPIA) database analysis

The differential expression of PRDX1 gene in normal cervical tissues and cervical cancer tissues is analyzed by using GEPIA database. GEPIA is a web server for analyzing the RNA sequencing expression data of 9,736 tumors and 8,587 normal samples from the The Cancer Genome Atlas and the Genotype Tissue Expression dataset projects, using a standard processing pipeline. GEPIA provides key interactive and customizable functions including differential expression analysis, profiling plotting, correlation analysis, patient survival analysis, similar gene detection, and dimensionality reduction analysis. GEPIA is available at http://gepia.cancer-pku.cn/.

### Cell lines and cell culture

The human cervical cancer cell line SiHa was obtained from Shanghai Cell Biology Medical Research Institute, Chinese Academy of Sciences, and cultured in Dulbecco's modified Eagle's medium (DMEM) (Gibco, Thermo Fisher Scientific, Waltham, MA, USA) supplemented with 10% fetal bovine serum (Gibco, Thermo Fisher Scientific), 100 U/mL penicillin and 100 μg/mL streptomycin (Gibco, Thermo Fisher Scientific). The cells were incubated at 37℃ in a humidified atmosphere of 5% CO_2_.

### Lentivirus construction and cell transfection

The cDNA and non-silencing short hairpin RNA for PRDX1 were synthesized by the company (Synbio Technologies, Suzhou, China) which were confirmed by sequencing, followed by inserted into the overexpression vector pLVX-IRES-ZsGreen 1 and knockdown vector PLKO.1, respectively. The recombinant plasmid was transfected into 293t cells together with packaging plasmids psPAX2 and G protein of the vesicular stomatitis virus (VSV-G) envelope plasmid pMD2.G (donated by Dr. Luzhe Sun, The University of Texas Health Science Center at San Antonio) to generate lentivirus.

Then SiHa cells were infected with lentivirus containing pLVX-PRDX1-IRES-ZsGreen 1 or empty vector to construct stable high expression or control cell line. To generate stable low expression cell line, SiHa cells were infected with lentivirus containing short hairpin RNA for PRDX1 or negative control vector. The efficiencies of overexpression and knockdown were determined by Western blot.

### Western blot analysis

Lentivirus infected SiHa cells were screened by puromycin (2 μg/ml) for two weeks. The cells were added with radioimmunoprecipitation assay buffer (Beyotime Biotechnology, Shanghai, China) to obtain the whole cell lysates. The protein concentration was quantified by bicinchoninic acid protein assay kit (Beyotime Biotechnology, Shanghai, China). Equal amounts of protein were added on each lane and were separated with 10 or 12% sodium dodecyl sulphate polyacrylamide gel electrophoresis. The proteins were then transferred onto polyvinylidene fluoride membrane (Millipore, Boston, MA, USA). The membranes were incubated with primary antibodies including PRDX1 (1:1000, Abcam, San Francisco, CA, USA), Nanog, PCNA, BAX, Bcl-2, Snail, E-cadherin, and MMP-9 (1:1000, Cell Signaling Technology, Beverly, MA, USA) overnight. After washed with Tris-buffered saline with tween 20, the membranes were incubated with the second antibody and detected with enhanced chemiluminescence reagent (Beyotime Biotechnology, Shanghai, China). Each experiment was repeated three times.

### Cell viability assay

Cervical cancer cells with stable knockdown and overexpression of PRDX1 or the corresponding control vector were seeded into 96-well plates at a concentration of 1000 cells each well. The Cell Counting Kit (CCK-8) reagent was added into the 96-well plates on 0, 1, 2, 3, 4 and 5 days, respectively. The absorbance was measured with a microplate reader at 450 nm. All experiments were performed with three independent trials.

### BrdU incorporation assay

Cervical cancer cells with stable knockdown and overexpression of PRDX1 or the corresponding control vector were seeded onto coverslips which were placed in the 6-well plate (1× 10^4^ cells/well) and bromodeoxyuridine (BrdU) was added 4 h before termination of cell culture. After that, the cells were fixed with 4% paraformaldehyde for 10 min at room temperature. Then endogenous peroxidase activity was inactivated with 0.3% hydrogen peroxide for 15 min at room temperature. RNase was added onto coverslips and incubated for 30 min at room temperature. The cells were then incubated with the mouse monoclonal antibody against BrdU (1:200, Cell Signaling Technology, Beverly, MA, USA) overnight at 4˚C. After washing with phosphate-buffered saline (PBS), the cells were labeled with Alexa Fluor 594-conjugated anti-mouse secondary antibodies (1:200, Thermo Fisher Scientific, Waltham, MA, USA). The nucleus was labeled with 4',6-diamidino-2-phenylindole (DAPI) (Thermo Fisher Scientific, Waltham, MA, USA). Olympus fluorescence microscope was used to capture fluorescent images. The total numbers of DAPI positive cells and total numbers of BrdU positive cells were counted from at least five images from each sample. Each experiment was repeated three times.

### Colony formation assay

For colony formation assay, cervical cancer cells were seeded into 6-well plates at a density of 400 cells per well and cultured in DMEM containing 10% fetal bovine serum for 2 weeks. The colonies were then fixed with methyl alcohol for 15 min and followed by staining with crystal violet for 10 min.

### Apoptosis assays

The effect of PRDX1 expression on cell apoptosis was assessed by using Annexin V-phycoerythrin (PE) and 7-amino-actinomycin D (7-AAD) apoptosis detection kit (BD, Franklin Lakes, NJ, USA) according to manufacturer's instructions. SiHa cells that have been infected with lentivirus containing PRDX1-cDNA or PRDX1-shRNA were collected and washed twice with cold PBS. Afterward, the cells were stained with 5 µL PE Annexin V and 5 µL 7-AAD for 15 min in the dark according to the manufacturer's protocol. The apoptotic cells were analyzed by CytoFLEX flow cytometer (Beckman Coulter). Three independent trials were performed.

### Scratch wound assay and transwell invasion assay

For the scratch wound assay, the cells were scratched with a plastic tip that was dragged across the cell monolayer upon cells reached confluence. Cell migration images were taken at 0 h and 24 h after scratching. Five fields were randomly selected, and the distances of migrated cells were measured under a light microscope. In invasion assay, cells were seeded into the upper chamber of a matrigel-coated transwell insert. Cells that did not invade were removed using a cotton swab and were stained by crystal violet and counted under an inverted microscope. Five random views were selected to count the cells.

### Nude mice xenograft model

Cervical cancer cells with overexpression or downregulation of PRDX1 and their corresponding control cells were digested and suspended in cold PBS at a concentration of 5 x 10^7^ cells/mL. Subsequently, the cells were subcutaneously inoculated into the back of the right forearm of 6-week-old female nude mice (100 μL every mouse). Two weeks after inoculation, the length and width of the tumor were measured every 3 days. The tumor volume was calculated as follows: V=length x width^2^/2. Tumors were harvested 5 or 6 weeks after inoculation, excised tumors were weighed and imaged with the camera. The study was approved by the Animal Research Ethics Committee of Wenzhou Medical University.

### Immunohistochemical analysis

Human cervical cancer tissues and tumors from xenograft model were fixed with formalin, embedded in paraffin and cut into 5-μm-thick sections. The slides were then deparaffinized with xylene, and rehydrated by using graded ethanol. Antigen retrieval was performed by using microwave heating for 20 min in sodium citrate-hydrochloric acid buffer. Then the sections were incubated with the anti-PRDX1 polyclonal rabbit antibody (1:100, Abcam, San Francisco, CA, USA) and anti-PCNA monoclonal mouse antibody (1:200, Cell Signaling Technology, Beverly, MA, USA) at 4°C overnight. Biotinylated goat anti-mouse or goat anti-rabbit antibody (ZSGB-BIO, Beijing, China) was used as a secondary antibody (20 min), followed by incubation with 3,3-diaminobenzidine tetrahydrochloride. Ultimately, the slides were counterstained with hematoxylin. For negative controls, PBS was substituted for the primary antibody.

The protein level of PRDX1 in tumor tissues and the paired adjacent non-tumor tissues was determined by semiquantitative IHC detection. The positively stained samples were scored as follows: 1, ≤25% of positive cells; 2, 25%-≤50% of positive cells; 3, 50%-≤75% of positive cells; 4, >75% of positive cells. The staining intensity was scored according to the following criteria: 0, negative staining; 1, weak staining; 2, moderate staining; and 3, strong staining. The final score was calculated by multiplying the percentage score by the staining intensity score.

### TUNEL assay

TUNEL assay was performed using the *in situ* cell death detection kit from Roche (Cat.No.11684817910) according to the manufacturer's instructions. Briefly, after being deparaffinized and rehydrated, the 5-μm-paraffin sections were permeabilized in 0.1% Triton X-100 for 8 min. The sections were then incubated with 50 μL TUNEL reaction mixture at 37℃ for 1 h. The samples were washed three times with PBS and the nucleus was stained with media containing DAPI. Olympus fluorescence microscope was used to capture fluorescent images. The total number of DAPI positive cells and TUNEL positive cells were counted from at least five images from each sample, respectively. Each experiment was repeated three times.

### Statistical analysis

Differences between two groups of cell growth curves and tumor growth curves of the xenograft model were analyzed by two-way ANOVA. The significance of other experiments was determined by using two-tailed unpaired Student's t-tests or one-way ANOVA. Results are expressed as mean ± SD from at least three independent experiments. *P* < 0.05 was considered to be statistically significant. All the statistical analyses were performed with Graph Pad Prism 6.0 software.

## Results

### The expression of PRDX1 is up-regulated in cervical cancer

**Figure 1:**
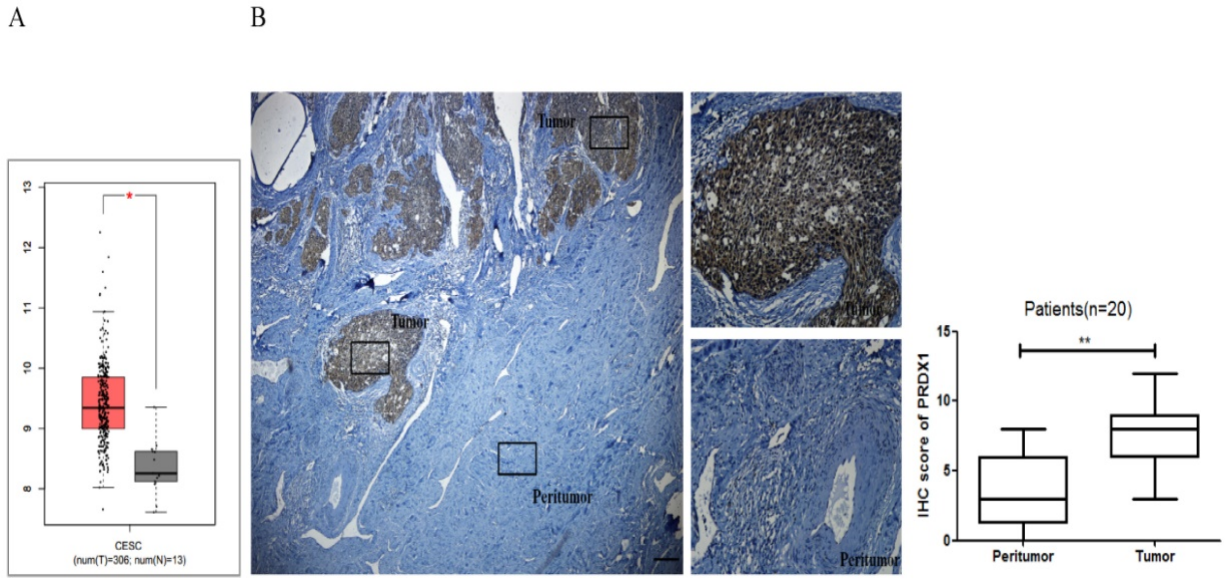
** The expression level of PRDX1 in human cervical cancer tissues.** (A) The GEPIA database revealed that PRDX1 expression was significantly up-regulated in cervical cancer tissues. (B) IHC staining images (left) and IHC scores (right) for PRDX1 expression in cervical cancer tissues and paired adjacent non-tumor tissues. Scale bar, 50 μm. *P <0.05, **P <0.01.

In this study, the mRNA expression of PRDX1 was investigated in the GEPIA database. As shown in Fig 1A, the expression of PRDX1 in 306 cervical cancer tissues was much higher than that in 13 noncancerous tissues. To validate this result, PRDX1 expression was further assessed in cervical cancer by immunohistochemical analysis of 20 cases of tissues including paired tumor and adjacent non-tumor tissues. Immunohistochemistry results showed that PRDX1 was mainly expressed in the nucleus and cytoplasm of cancer cells (Fig 1B). PRDX1 was found abundantly expressed in tumor tissues, while was rarely expressed in paired adjacent non-tumor tissues (Fig 1B *left*). The immunohistochemical scores of the two groups were statistically significant (Fig 1B *right*). Next, patients were divided into PRDX1 low and PRDX1 high group according to the result of IHC staining, and the correlation between the PRDX1 expression and clinicopathological parameters was investigated. We found that sixty percent of patients exhibited high level of PRDX1 and the level of PRDX1 was significantly associated with tumor stage, lymphatic metastasis and differentiation, but it was not associated with age (Table 1). These results suggest that PRDX1 may frequently overexpress in human cervical cancer.

### Lentivirus-mediated upregulation and downregulation of PRDX1 in SiHa cells

**Figure 2:**
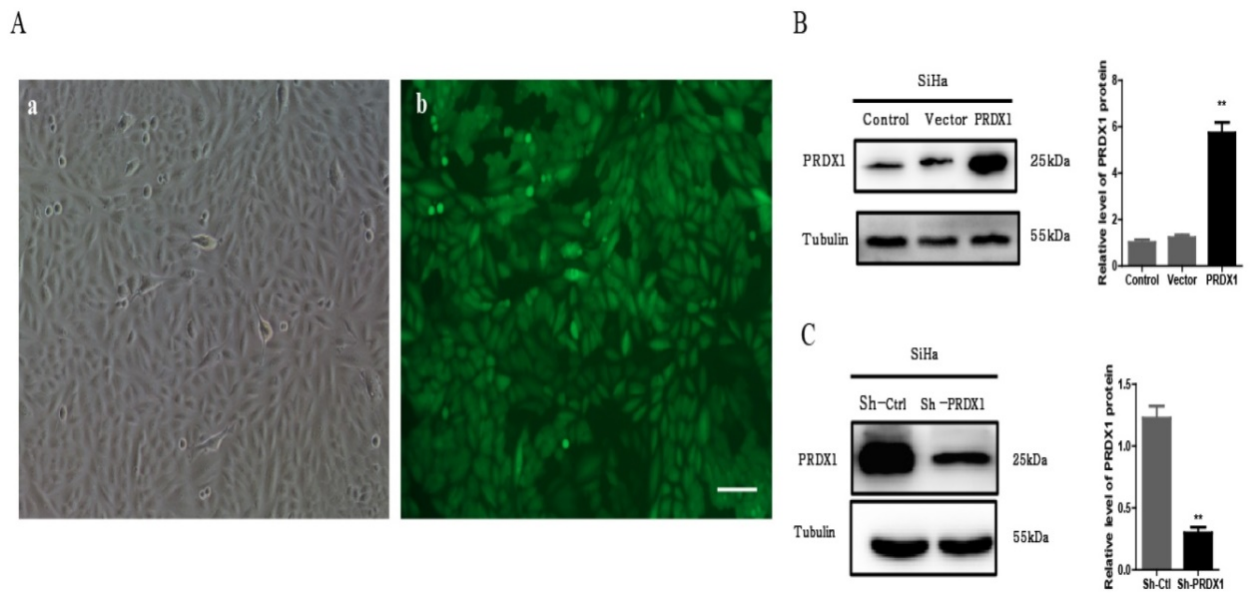
** Establishment of PRDX1 overexpressed and knockdown cervical cancer cell models.** (A) Infection efficiency detected by fluorescence microscopy 48 h after lentivirus infection. a: image of SiHa cells at normal light. b: GFP expression in SiHa cells following transfection with lentivirus. Scale bar, 50 μm. (B) Lentivirus containing PRDX1-pLVX-IRES-ZsGreen dramatically upregulated the expression of PRDX1 in Siha cells. (C) Lentivirus containing shRNA against PRDX1 (Sh-PRDX1) significantly reduced the PRDX1 expression in SiHa cells. **P <0.01, data were expressed as mean ± SD from triplicate experiments.

To investigate the biological functions of PRDX1 in cervical cancer, SiHa cells were infected with lentivirus containing PRDX1-cDNA or PRDX1-shRNA to stable up-regulate or down-regulate the expression of PRDX1. The infection efficiency of lentivirus is shown in Fig 2A. As shown by Western blot analysis, PRDX1-cDNA containing lentivirus significantly increased the expression of PRDX1 in SiHa cells (Fig 2B). SiHa cells infected with lentivirus containing PRDX1-shRNA showed reduced expression of PRDX1 when compared with corresponding control cells (Fig 2C).

### PRDX1 overexpression promotes the proliferation of cervical cancer cells

**Figure 3:**
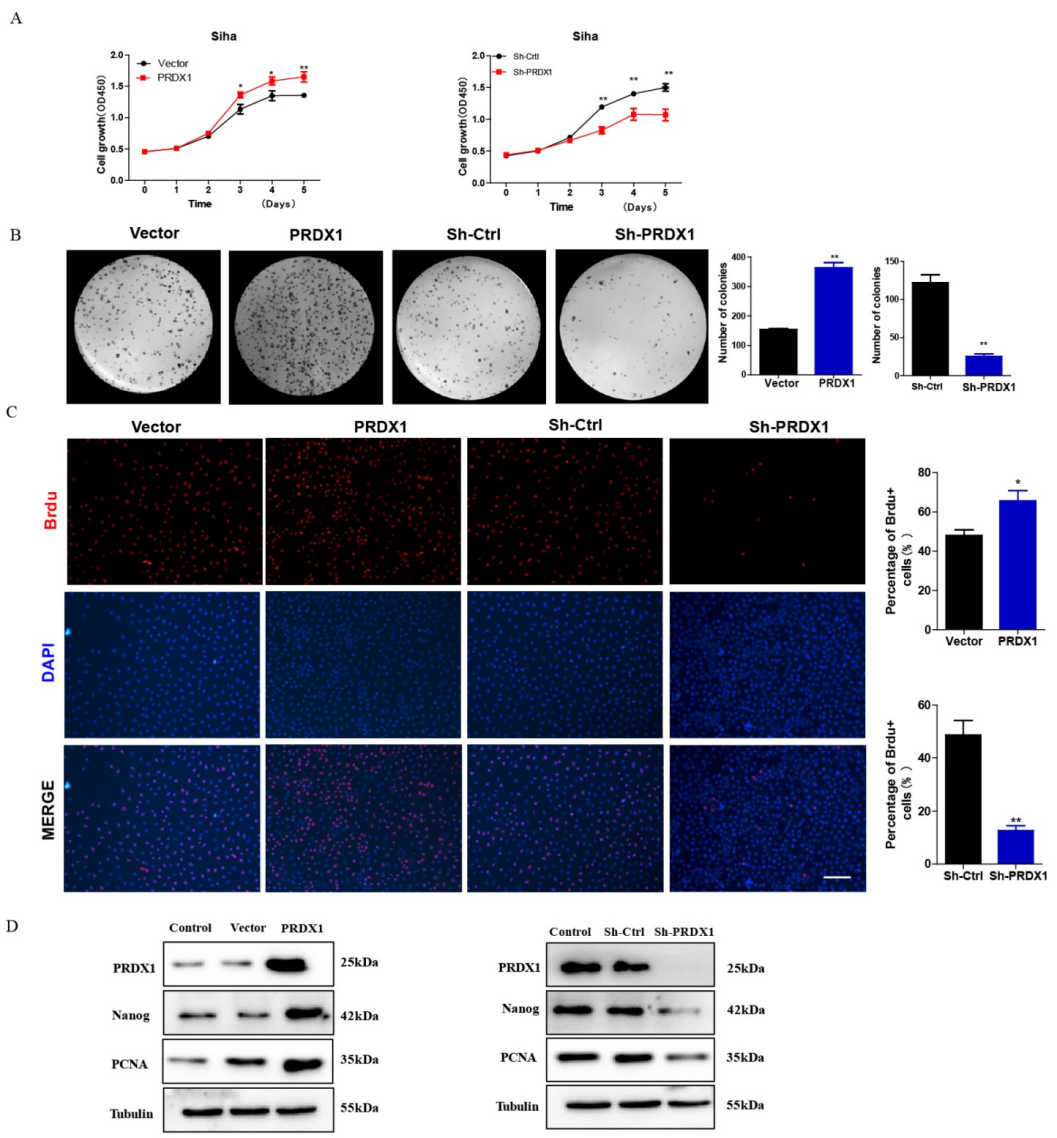
** PRDX1 promotes cervical cancer cell proliferation *in vitro*.** (A) PRDX1 overexpressed SiHa cells grow faster than control cells and PRDX1 knockdown impaired the growth of SiHa cells. Cells infected with lentivirus containing PRDX1 -shRNA or PRDX1-cDNA were cultured in a 96-well plate for 5 days. Cell viability was determined using the CCK-8 reagent. (B) Colony formation assay in PRDX1 overexpressed and PRDX1 knockdown SiHa cells. (C) Cell proliferation ability was determined using BrdU incorporation assay. Scale bar, 50 μm. (D) The expression of PRDX1, Nanog and PCNA in PRDX1 overexpressed or knockdown SiHa cells were detected by Western blot analyses. * P <0.05, ** P <0.01, data were expressed as mean ± SD from triplicate experiments.

To investigate whether PRDX1 act as a cancer-promoting gene in cervical cancer, PRDX1 overexpressed or knockdown cells were subjected to growth analyses. CCK-8 assay revealed that overexpression of PRDX1 significantly promoted cell growth (Fig 3A). We also observed that knockdown of PRDX1 inhibited cell growth (Fig 3A). Colony formation assays showed that overexpression of PRDX1 caused an increase of colony numbers compared with control cells, the opposite result was observed in cells with PRDX1 knockdown (Fig 3B). BrdU incorporation assay revealed that the percentage of BrdU positive cells were increased in PRDX1 overexpressed SiHa cells and were significantly decreased in PRDX1 knockdown SiHa cells (Fig 3C). We further examined whether PRDX1 affected the expression of proliferation-related protein. The results showed that overexpression of PRDX1 up-regulated the expression of PCNA and stem-cell-abundant protein Nanog (Fig 3D). The expressions of PCNA and Nanog were significantly inhibited when PRDX1 was suppressed (Fig 3D). These results indicate that PRDX1 promotes the proliferation of cervical cancer cells possibly via upregulation of PCNA and Nanog.

### PRDX1 overexpression decreases the apoptosis of cervical cancer cells

**Figure 4:**
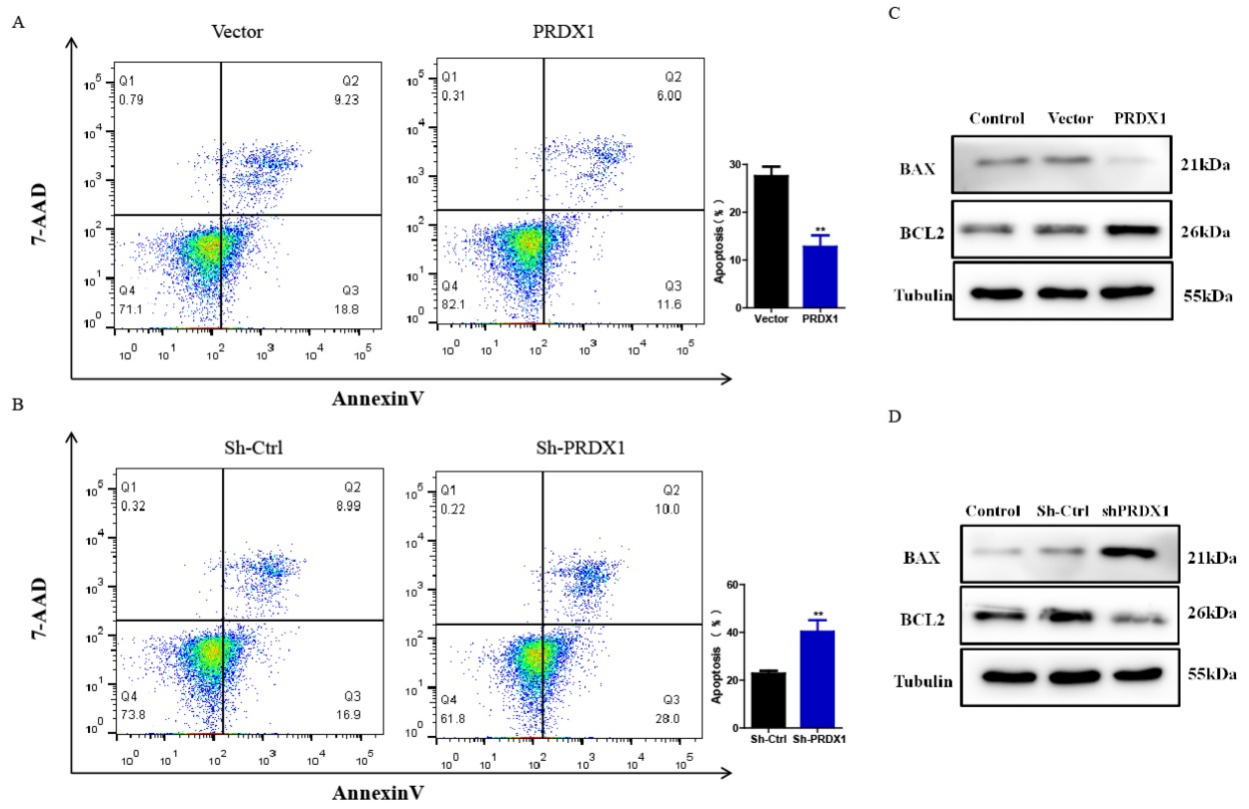
** PRDX1 inhibits apoptosis of cervical cancer cells.** (A) PRDX1 overexpressed and control SiHa cells were collected, and the apoptosis of cells was analyzed by Flow cytometry. (B) PRDX1 knockdown and control SiHa cells were collected and the apoptosis of cells were analyzed by Flow cytometry. (C and D) The expression of BAX and Bcl-2 in PRDX1 overexpressed or knockdown SiHa cells were detected by Western blot analyses. ** P <0.01, data were shown as mean ± SD from triplicate experiments.

Next, whether PRDX1 was involved in cervical cancer cell apoptosis was examined. Our results showed that PRDX1 overexpression inhibited while PRDX1 knockdown promoted apoptosis. The average percentage of apoptotic cells was 12.81% in SiHa cells with PRDX1 overexpression versus 27.62% in vector control cells; 40.33% in PRDX1 downregulated SiHa cells versus 22.76% in Sh-control cells (Fig 4A and 4B). On the basis of the antiapoptotic effect of PRDX1, we further investigated whether PRDX1 could regulate the expression of apoptosis-related protein. Our results showed that overexpression of PRDX1 significantly enhanced the expression of Bcl-2 and inhibited the expression of BAX (Fig 4C). In contrast, knockdown of PRDX1 significantly increased BAX expression and decreased Bcl-2 expression in SiHa cells (Fig 4D). These findings indicate that PRDX1 participates in the regulation of cervical cancer cell apoptosis.

### PRDX1 overexpression promotes invasion and migration of cervical cancer cells

**Figure 5:**
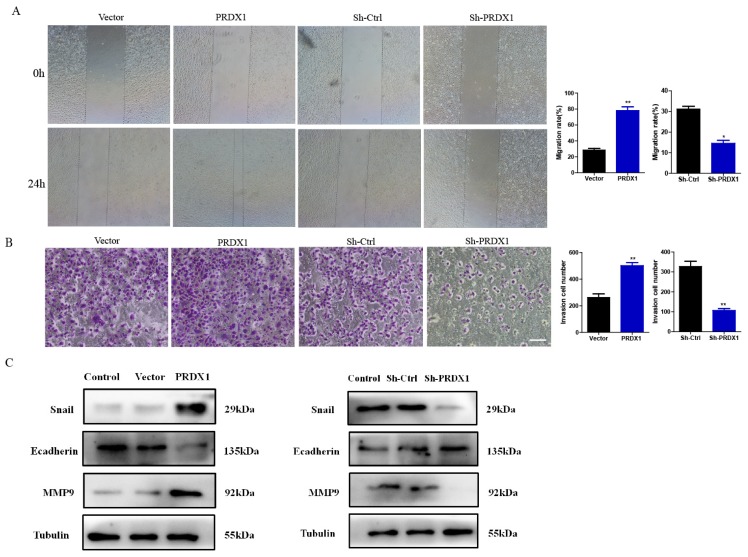
** PRDX1 promotes cervical cancer cell migration and invasion.** (A) The relative cell migration ratio was increased in PRDX1 overexpressed SiHa cells and suppressed in PRDX1 knockdown SiHa cells. (B) The relative cell invasion ratio was increased in PRDX1 overexpressed SiHa cells and suppressed in PRDX1 knockdown SiHa cells. Scale bar, 50 μm. (C) The expression of MMP-9, Snail and E-cadherin in PRDX1 overexpressed or knockdown SiHa cells were detected by Western blot analyses. * P <0.05, ** P <0.01, data were shown as mean ± SD from triplicate experiments.

To investigate the role of PRDX1 in the processes of cervical cancer cell migration and invasion, SiHa cells with PRDX1 overexpression or downregulation were subjected to wound healing and transwell assays. As shown in Fig 5A, the ability of migration was improved in cells with overexpression of PRDX1 when compared to control cells. Whereas, knockdown of PRDX1 dramatically inhibited cell migration (Fig 5A). Transwell invasion assay demonstrated that overexpression of PRDX1 significantly increased the invasive ability of SiHa cells (Fig 5B). Instead, knockdown of PRDX1 markedly reduced the number of invasive SiHa cells when compared with the Sh-Ctrl control group (Fig 5B). Additionally, we examined the expression of MMP family proteins and epithelial-mesenchymal transition (EMT) related proteins. The results showed that overexpression of PRDX1 in SiHa cells up-regulated Snail and MMP-9 protein levels and down-regulated E-cadherin protein level (Fig 5C). The opposite results were observed in PRDX1 knockdown group (Fig 5C).

### PRDX1 overexpression promotes tumor growth of cervical cancer cells *in vivo*

**Figure 6:**
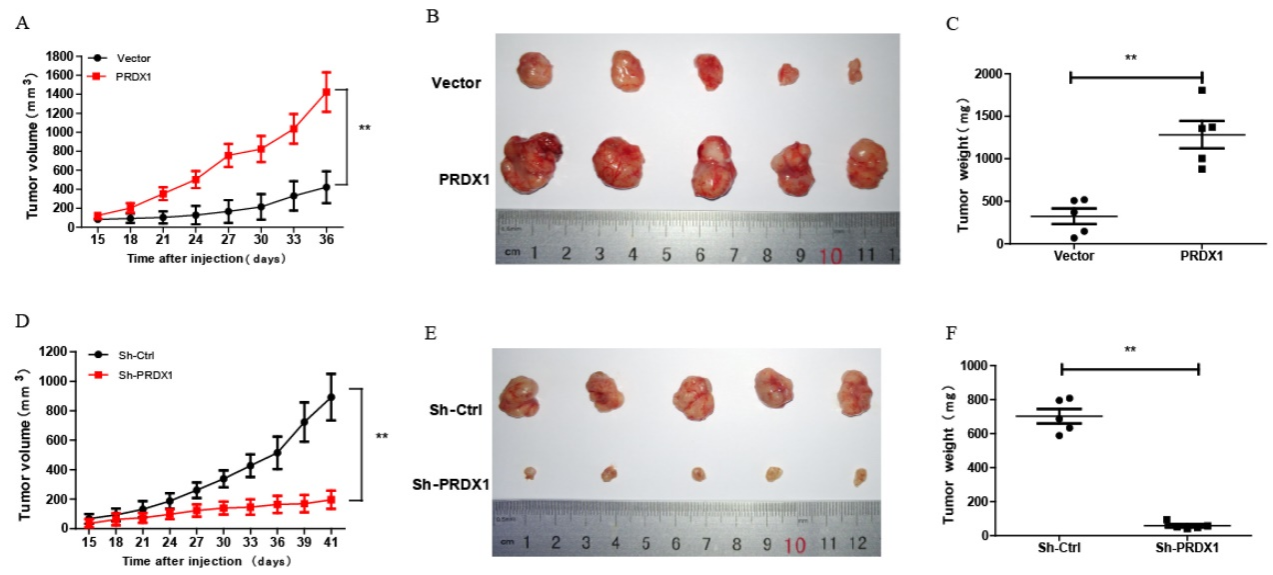
** PRDX1 promotes cervical cancer cell growth* in vivo*.** (A and D) Tumor volume of subcutaneous xenograft tumor model developed from PRDX1 overexpressed SiHa cells and PRDX1 knockdown SiHa cells. The length (L) and width (W) of the xenograft tumor were measured every 3 days. The tumor volumes were calculated according to the formula (L ×W^2^)/2. (B and E) Xenograft tumor were dissected from the mice and put together to take pictures after the mice were sacrificed. (C and F) The tumor weight was determined after tumor nodules were harvested. ** *P* <0.01, data were shown as mean ± SD from triplicate experiments.

**Figure 7:**
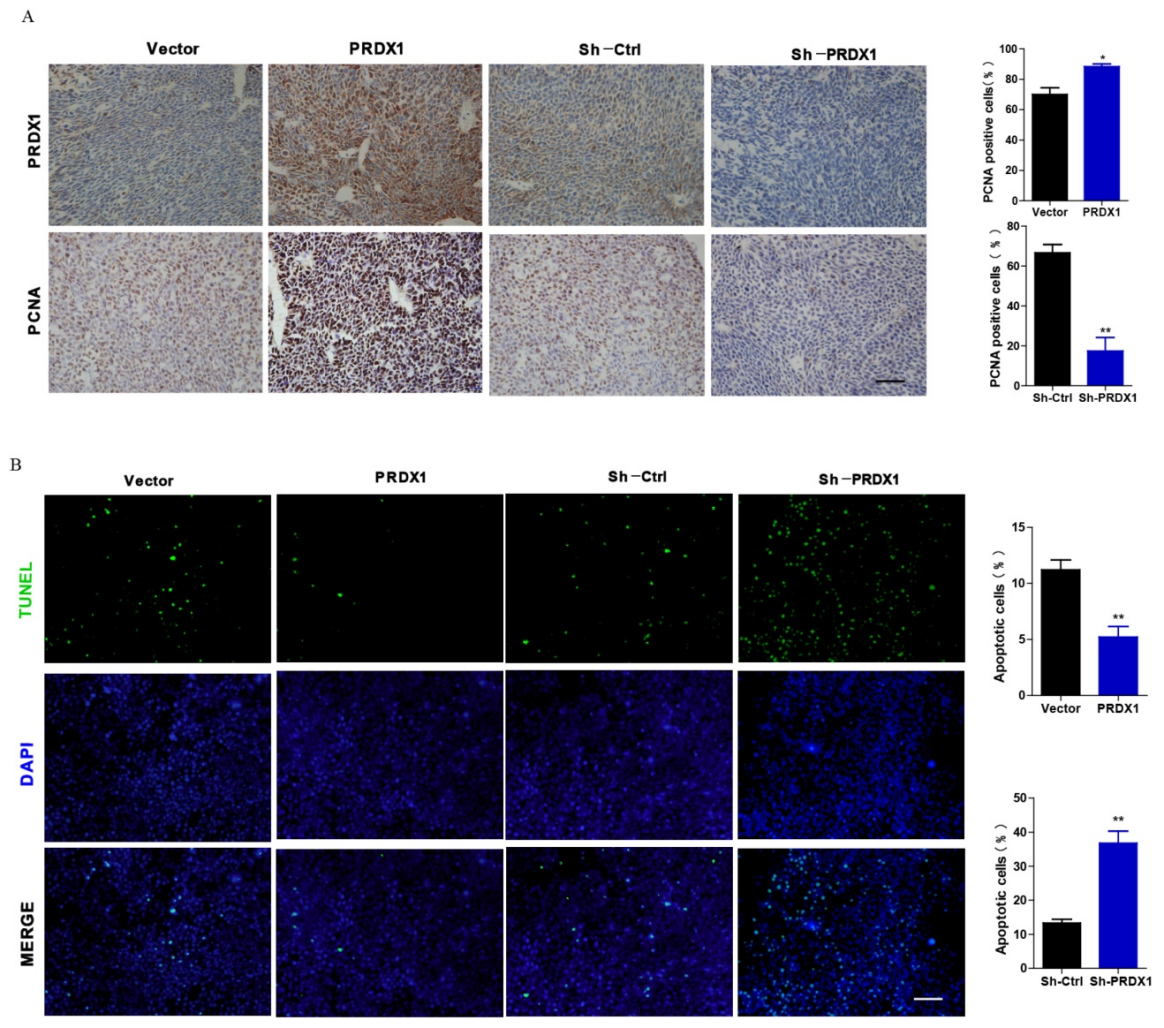
** Representative immunohistochemical staining of PRDX1 and PCNA and TUNEL staining images in xenograft tumor.** (A) Tumor tissues developed from PRDX1 overexpressed or PRDX1 knockdown SiHa cells were analyzed by PRDX1 and PCNA staining. Scale bar, 50 μm. (B) Tumor tissues developed from PRDX1 overexpressed or PRDX1 knockdown SiHa cells were analyzed by TUNEL staining. Scale bar, 50 μm. * *P* <0.05, ** *P* <0.01, data were shown as mean ± SD from triplicate experiments.

To explore whether PRDX1 acts as a cancer-promoting gene *in vivo*, xenograft tumor model was carried out by injecting nude mice with PRDX1 stable overexpression or knockdown SiHa cells. Exogenous overexpression of PRDX1 significantly promoted tumor growth, whereas the growth of the tumor was severely suppressed by knockdown of PRDX1 in SiHa cells (Fig 6A, 6B, 6D and 6E). Moreover, those xenografts grew from SiHa cells with PRDX1 overexpression weighted much heavier compared with those from the control cells (Fig 6C). On the contrary, knockdown the expression of PRDX1 in SiHa cells resulted in a significant decrease in tumor weight (Fig 6F). Xenografts developed from SiHa cells with PRDX1 stable knockdown showed a considerable decrease of positive PCNA staining and increase of positive TUNEL staining apoptosis cells (Fig 7A and 7B). In contrast, overexpression of PRDX1 significantly increased PCNA-positive staining and decreased TUNEL-positive staining in xenografts developed from SiHa cells (Fig 7A and 7B). These findings confirm that PRDX1 promotes tumor cell growth *in vivo* by promoting cell proliferation and inhibiting cell apoptosis.

## Discussion

In recent years, there has been an increasing interest in exploring the role of PRDXs in cancerogenesis. Amongst the PRDX family members, PRDX1 possesses the widest cellular distribution and shows the highest abundance in various tissues 6. PRDX1 has been found to up-regulated in multiple cancer tissues. Previous studies have shown that PRDX1 facilitates the infiltrative growth of isocitrate dehydrogenase-wildtype gliomas by forming a heterodimer with p38α 24. On the contrary, PRDX1 was also confirmed to be a tumor suppresser by protecting PTEN from oxidation-induced inactivation 22. Until now, the change in PRDX1 expression during the process of cervical cancer carcinogenesis is unclear. To unravel this confusion, the expression of PRDX1 in the tissue from cervical cancer patients was detected by immunohistochemistry staining. Our study showed that PRDX1 abundantly expressed in tumor tissues but not in corresponding para-carcinoma tissues.

The effects of PRDX1 overexpression on cervical cancer have not been investigated thoroughly. To further investigate the function of PRDX1 in cervical cancer progression, we used recombinant lentivirus to upregulate or downregulate the expression of PRDX1. Our study, for the first time, showed that down-regulation of PRDX1 suppressed cervical cancer cell growth as demonstrated by the reduced growth speed and decreased the proportion of BrdU-positive cells compared with the control cells. Our presented data also showed that knockdown of PRDX1 dramatically inhibited cell migration and invasion. Besides, the promoting effect of PRDX1 on cellular growth and progression was verified by subcutaneous transplanted model.

Some recent studies have shown that PRDX1 contributes to the progression of diverse cellular processes via regulating different signaling pathways in multiple types of tumors. In osteosarcoma cells, PRDX1 promotes cell proliferation and metastasis through regulating Akt/mammalian target of rapamycin (mTOR) signaling pathway 25. PRDX1 promotes prostate cancer growth and progression by increasing the expression of the vascular endothelial growth factor 26. Consistent with the previous findings, our study demonstrated that overexpression of PRDX1 significantly promotes cervical cancer cell proliferation, and enhanced the expression of Nanog, PCNA and Bcl-2 and decreased the expression of BAX. We hypothesized that PRDX1 regulated the process of cervical cancer cell proliferation via upregulating the expression of Nanog and PCNA. In addition, PRDX1 affects the process of cellular apoptosis by regulating the expression of apoptosis-related protein Bcl-2 and BAX.

Alterations in the expression of proteins related to the intrinsic apoptotic pathway have been associated with various types of human cancers. The intrinsic pathway mediated apoptosis is controlled by the Bcl-2 family of anti-apoptotic members (such as Bcl-2 and Bcl-xL) and pro-apoptotic members (such as Bad, Bid, BAX and Bim) 27. Disruption of the balance of these antagonistic pro- and anti-apoptotic Bcl-2 family members, which counteract the activity of each another, mediate the occurrence of apoptosis 28. Bcl-2 promotes survival of tumor cells by binding to pro-apoptotic BAX subfamily members. Overexpression of Bcl-2 has been reported to be involved in a variety of human malignancies through inhibiting apoptosis and accelerating tumorigenesis 29,30. BAX, as a proapoptotic member of Bcl-2 family members, counteracts the anti-apoptotic effect of Bcl-2 protein to promote apoptosis of cells 31. The expression of BAX, one of the major apoptosis-related biomarkers, correlated with development, progression and prognosis of a variety of malignant tumors 32. Apoptosis is induced when BAX forms a homodimer and apoptosis is inhibited when BAX forms a heterodimer with Bcl-2. Now our research showed that down-regulation of PRDX1 resulted in reduced expression of Bcl-2 and increased expression of BAX. This may be one of the mechanisms by which PRDX1 inhibits the apoptosis of tumor cells and participates in cervical cancer tumorigenesis.

Nanog, a divergent homeobox domain protein, is a key transcription factor involved in maintaining the self-renewal and pluripotency of embryonic stem cells 33. Accumulated evidence suggests that Nanog confers cancer cells certain cancer stem cells properties and promotes immortalization of the entire tumor population by empowering subsets of cancer cells with self-renewal potential 34. Aberrant overexpression of Nanog has been observed in squamous cervical carcinomas 35. One study reported that Nanog presented in cervical tissues and its expression was associated with the progression of cervical cancer 35. Previous studies have also pinpointed that increased expression of PCNA is associated with poor 5-year survival and advanced pathological stage in cervical cancer 36. In the present study, we found that upregulation or downregulation the expression of PRDX1 significantly promoted or suppressed the proliferation and colony formation of cervical cancer cells. Moreover, the expressions of Nanog and PCNA were up-regulated in PRDX1 overexpressed SiHa cells and were downregulated in PRDX1 knockdown SiHa cells. Collectively, we proposed that PRDX1 promoted tumor cell proliferation probably through regulating Nanog and PCNA expression.

In colorectal cancer, gastric cancer and lung cancer, PRDX1 has been proven to promote tumor metastasis, but PRDX1 has also been pointed out to act as a tumor metastasis suppressor in hepatocellular carcinoma and osteosarcoma^12^,^15^,^37^-^39^. In our study, PRDX1 was found to significantly promoted the migration and invasion of cervical cancer cells. These studies indicate that PRDX1 plays different roles in tumor metastasis. Furthermore, the role of PRDX1 in promoting tumor metastasis was associated with increased expression of Snail and MMP-9 and decreased expression of E-cadherin. Snail, as an important mediators of EMT, shows increased level in many malignant tumors, and the expression of which is associated with invasiveness and metastatic potential of tumors 40,41. The role of Snail in cancer cell metastasis is partly mediated through repressing the transcription of cell adhesion molecule E-cadherin 42. For example, Snail was reported to inhibit transcription of E-cadherin by interacting with Suv39H1 (suppressor of variegation 3-9 homolog 1) in breast cancer 43. E-cadherin is an important protein for adhesion between cells in epithelial tissues, and loss of E-cadherin will lead to enhanced invasion and migration of various tumor cells 44. Besides, Snail can also promote cancer cell invasion through upregulating the expression of matrix metalloproteinases 45. It was reported that blocking Snail expression inhibited the activity of MMP-9 in ovarian cancer cells 46. MMP-9 belongs to the family of matrix metalloproteinases, and its expression is positively correlated with the invasive potential of cervical cancer 47. Here, we detected an increased expression of Snail and MMP-9 decreased expression of E-cadherin when PRDX1 was overexpressed. Accordingly, we speculate that PRDX1 probably facilitates migration and invasion via up-regulating the expression of Snail, which further inhibits E-cadherin expression and upregulates the expression of MMP-9. E-cadherin down-regulation and MMP-9 up-regulation induced by Snail can further accelerate the invasion and migration of cervical cancer cells. Although further studies are necessary to confirm our speculation, the stimulative effect of PRDX1 on cervical cancer cell migration and invasion is of particular interest.

In this study, the effect of PRDX1 expression on the proliferation and metastasis of cervical cancer was studied on SiHa cells. The results should be further verified on other cervical cancer cell lines. In addition, further study is needed to investigate the *in vivo* effect of PRDX1 expression on the metastasis of cervical cancer cells. This study indicates that PRDX1 overexpression promotes proliferation and metastasis of cervical cancer. However, the detailed mechanism underlying PRDX1 overexpression in cervical cancer remains unclear and worthy of further study.

To conclude, our study indicates that as an oncogene, PRDX1 is an important regulator of cervical cancer which facilitates tumor cell proliferation, migration and invasion and suppresses cellular apoptosis. Furthermore, we found that PRDX1 exerted its carcinogenic potential by regulating Nanog, PCNA, Bcl-2, BAX and Snail expression. Taken together, our results suggest PRDX1 function as an oncogene and PRDX1-specific inhibitor may be a promising drug for target therapy in patients with cervical cancer.

## Figures and Tables

**Table 1:** Association of PRDX1 expression with clinicopathological parameters of cervical cancer patients.

Clinicopathological Parameters	Cases	PRDX1 expression		*P* value
		low	high	
**Age (years)**				>0.05
>50 <50	8 12	4 4	4 8	
**Stage**				<0.05
Ia+ Ib IIa+ IIb	11 9	6 2	5 7	
**Differentiation**				<0.01
Well moderately Poorly	15 5	7 1	8 4	
**Lymphatic metastasis**				<0.05
Absent Present	14 6	6 2	8 4	

## Abbreviations

PRDX1: peroxiredoxin 1
NAC: neoadjuvant chemotherapy
IHC: immunohistochemistry
GEPIA: Gene expression profiling interactive analysis
DMEM: Dulbecco's modified Eagle's medium
VSV: vesicular stomatitis virus
CCK: Cell Counting Kit
BrdU: bromodeoxyuridine
PBS: phosphate-buffered saline
PE: phycoerythrin
7-AAD: 7-amino-actinomycin D
TUNEL: TdT-mediated dUTP nick end labeling
EMT: epithelial-mesenchymal transition
ROS: reactive oxygen species
MMP-9: matrix metallopeptidase 9
