# Up-regulation of peroxiredoxin-1 promotes cell proliferation and metastasis and inhibits apoptosis in cervical cancer: Erratum

**DOI:** 10.7150/jca.74424

**Published:** 2022-05-28

**Authors:** Ermei Lu, Xiaoli Hu, Chunyu Pan, Jingjing Chen, Yichi Xu, Xueqiong Zhu

**Affiliations:** Department of Obstetrics and Gynecology, the Second Affiliated Hospital of Wenzhou Medical University, Wenzhou 325027, China.

The authors recently noticed an inadvertent error in the initially published version of our article and would like to correct it.

Due to our negligence, an incorrect image of TUNEL staining was selected for PRDX1 group in Figure 7B when combining different images into a figure. After proofreading, the correct picture has been provided to show the TUNEL positive cells in tumor tissue of PRDX1 group in Figure 7B.

The correct Figure 7 is shown below. This correction does not affect the results and conclusions of the study. The authors apologize for any inconvenience the error may have caused.

## Figures and Tables

**Figure 7:**
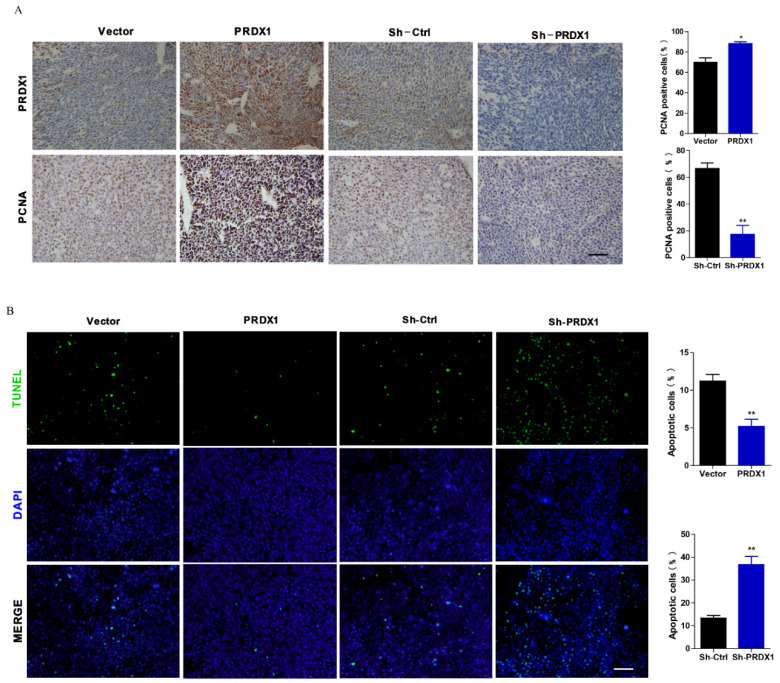
The corrected new figure.

